# Dysbiosis of Fecal Microbiota From Complement 3 Knockout Mice With Constipation Phenotypes Contributes to Development of Defecation Delay

**DOI:** 10.3389/fphys.2021.650789

**Published:** 2021-07-19

**Authors:** Yun Ju Choi, Ji Eun Kim, Su Jin Lee, Jeong Eun Gong, Hong Joo Son, Jin Tae Hong, Dae Youn Hwang

**Affiliations:** ^1^Department of Biomaterials Science (BK21 FOUR Program), College of Natural Resources and Life Science/Life and Industry Convergence Research Institute, Pusan National University, Miryang, South Korea; ^2^Department of Life Science and Environmental Biochemistry, College of Natural Resources and Life Science/Life and Industry Convergence Research Institute, Pusan National University, Miryang, South Korea; ^3^College of Pharmacy, Chungbuk National University, Cheongju, South Korea; ^4^Laboratory Animals Resources Center, Pusan National University, Miryang, South Korea

**Keywords:** complement C3, constipation, dysbiosis, fecal microbiota transplantation, C-kit

## Abstract

Significant phenotypes for constipation were detected in complement 3 (C3) knockout (KO) mice, although no research has been conducted on an association with alteration of gut microbiota. To investigate the effects of dysbiosis on fecal microbiota from C3 KO mice with constipation, the composition of fecal microbiota was characterized in mid-colons of 16-week-old C3 KO mice, and their function for defecation delay development was examined after fecal microbiota transplantation (FMT) of C3 KO mice. Some significant alterations in constipation phenotypes, including stool parameters and histopathological structure, were detected in 16-week-old C3 KO mice compared to those of wild-type (WT) mice. Fecal microbiota of C3 KO mice exhibited decreases in *Anaerocolumna*, *Caecibacterium*, *Christensenella*, *Kineothrix*, and *Oscillibacter* populations and increases in *Prevotellamassilia*, *Reuthenibacterium*, *Prevotella*, *Eubacterium*, *Culturomica*, *Bacteroides*, and *Muribaculum* populations. In FMT study, key stool parameters, including weight and water content, were remarkably declined in a transplanted KO (KFMT) group of antibiotics-induced depletion of microbiota (AiDM)-WT and AiDM-KO mice, and a similar change was observed in fecal morphology. However, intestine length decreased in only the KFMT group of AiDM-WT mice compared with that of AiDM-KO mice. The mucosal layer and muscle thickness were commonly decreased in the KFMT group of AiDM-WT and AiDM-KO mice, and significant alterations in the crypt structure of Lieberkuhn and molecular regulators, including AQP8, C-kit, and 5-HT, were observed in the same group. Taken together, results of the present study indicate that dysbiosis of fecal microbiota from C3 KO mice with constipation phenotypes has a key role in the induction and regulation of defecation delay.

## Introduction

Dysbiosis has been defined as a homeostatic imbalance of the gut microbiota due to disturbance of the flora, its functional composition, and metabolic activity, as well as alteration of local distribution during antibiotic use, psychological and physical stresses, radiation, altered gastrointestinal tract peristalsis, and dietary changes (Hawrelak and Myers, 2004; Bien et al., 2013; Knights et al., 2013). Such imbalance has been classified into three types: loss of microorganism diversity, loss of beneficial microorganisms, and overgrowth of harmful microorganisms, although the authors did not cover all causes (DeGruttola et al., 2016). Also, dysbiosis is implicated in various chronic diseases, such as inflammatory bowel disease (IBD), obesity, diabetes, autism spectrum disorders, cancer, allergy, and periodontal disease (Carding et al., 2015; DeGruttola et al., 2016). During the pathogenesis of these diseases, microbiota composition can significantly change; populations of some pathogens increase, while others decrease. In IBD, an increase of three major pathogens, *Mycobacterium avium* subsp. *paratuberculosis*, *Escherichia coli*, and *Clostridium difficile*, as well as a decrease in two bacteria groups, Firmicutes and Bacteroidetes, have been frequently detected in the intestines of patients (Clayton et al., 2009; Li et al., 2015). Therefore, dysbiosis has been considered as a target for treatment strategies, including antibiotics treatments, fecal microbiota transplantation (FMT), and probiotics administration, in several related diseases (Kim et al., 2006; Sharara et al., 2006; Smith et al., 2014).

Meanwhile, some scientific evidence indicates that dysbiosis of intestinal microbiota may contribute to the pathogenesis and related symptoms of chronic constipation (Choi and Chang, 2015; Zhao and Yu, 2016). However, there are significant discordances among the alterations of intestinal microbiota from patients with chronic constipation. The levels of some microaerophilic or obligate anaerobic bacteria, including *Lactobacillus*, *Bifidobacterium*, and *Bacteroides* spp., decrease in patients with chronic constipation, while the levels of *Pseudomonas aeruginosa* and *Campylobacter jejuni* increase in the same patient (Kirgizov et al., 2001; Khalif et al., 2005; Gerritsen et al., 2011; Kim et al., 2015). In patients with constipation predominant-irritable bowel syndrome (IBS-C), the levels of *Bacteroides* spp. and Enterobacteriaceae were increased, while the population levels of *Bifidobacteria*, *Clostridium leptum*, and *Faecalibacterium prausnitzii* were decreased (Simren et al., 2013; Nourrisson et al., 2014). A significant increase in the levels of *Bifidobacteria* and *Clostridium leptum* was detected in feces of constipated children (Zoppi et al., 1998). Other constipated patients show a decreased abundance of *Prevotella* and an increased level of Firmicutes, although *Lactobacillus* and *Bifidobacterium* maintained a constant level (Zhu et al., 2014). Some butyrate-producing genera, including *Coprococcus*, *Roseburia*, and *Faecalibacterium*, display increased levels in constipated patients (Pryde et al., 2002; Sokol et al., 2008). Therefore, further research is required to provide scientific evidence elucidating the correlation between dysbiosis and chronic constipation.

In this study, we characterized alterations in the fecal microbiota of C3 KO mice with constipation phenotypes, and the role of their fecal dysbiosis on defecation delay was verified in an FMT study that transferred fecal of C3 KO mice into WT mice.

## Materials and Methods

### Management of Animal Study

The study protocol for the animal study was approved by the Pusan National University-Institutional Animal Care and Use Committee (PNU-IACUC; approval number PNU-2019-2292). All mice were handled at the Pusan National University-Laboratory Animal Resources Center, which is accredited by the Korea Food and Drug Administration (FDA; Accredited Unit Number: 000231) and AAALAC International (Accredited Unit Number: 001525). Adult mice were provided, *ad libitum*, with standard irradiated chow diet (Samtako BioKorea Co., Osan, Korea) consisting of moisture (12.5%), crude protein (25.43%), crude fat (6.06%), crude fiber (3.9%), crude ash (5.31%), calcium (1.14%), and phosphorus (0.99%). These animals were maintained in a specific pathogen-free state under a strict light cycle (lights on at 06:00 h and off at 18:00 h) at 22 ± 2°C and relative humidity 50 ± 10%.

### Experimental Design of C3 KO Mice

Eight-week-old male C3 KO mice with CRISPR/Cas9-mediated C3 mutant genes and wild-type (WT) FVB/N background strains were kindly provided by the Department of Laboratory Animal Resources (Laboratory Animals Resources Bank) at the National Institute of Food and Drug Safety Evaluation (NIFDS, Chungju, Korea). After breeding and maintenance for up to 16 weeks, the wild-type (WT, *n* = 7) and KO (*n* = 7) mice were used in the analysis of constipation. Subsequently, all 16-week-old mice were euthanized using a chamber filled with CO_2_ gas, and their mid-colons were subsequently harvested for additional analysis.

### Feeding Behavior and Stool Parameters Analyses

The food weight, water volume, and body weight were measured in 16-week-old WT and C3 KO mice using an electronic balance (for food and body weight) and a measuring cylinder (for water volume) as described in previous study (Park et al., 2021). Also, three stool parameters, including weight, number, and moisture content, were measured in mice of subset groups because stool consistency strongly relates to stool transit time and defecation frequency. To achieve these, each mouse of subset group was held in individual metabolic cages (Daejong Ltd., Seoul, Korea) for 12 h in order to avoid contamination of stools. Stools excreted from each mouse were collected at 10:00 a.m. Stool weight was measured three times per sample using an electric balance (*Mettler Toledo*, Columbus, OH, United States), and the number of stools was counted twice per animal. Stool moisture content was estimated by applying the following calculation method:

$$\mathrm{Stool moisture content}=\left(A-B\right)/A\times 100,$$

where A is the weight of fresh stools collected from mice, and B is the weight of stools after drying at 60°C for 24 h. Furthermore, urine volume collected at 9 a.m. on the next day was measured twice per sample using a cylinder.

### Histopathological Analysis

Generally, the colon is well known to play an important role in the formation and transit of stools because it absorbs water and some nutrients from the digested waste materials as well as compacts and storage stools in the rectum until defecation (Andrews and Storr, 2011; Bendezú et al., 2017). The histopathological structure of colon was remarkably changed in various constipation animals although these structural changes were recovered with treatment of therapeutic drugs and natural products (Kim et al., 2019a; Zhao et al., 2019). Therefore, mid-colon was selected as target tissue based on various previous studies (Andrews and Storr, 2011; Kim et al., 2019a). Mid-colons were prepared for histological analysis as previously described (Kim et al., 2019b). Briefly, mid-colons harvested from a subset group of mice were fixed with 10% formalin for 12 h, embedded in paraffin wax, sectioned to 4 μm thick slices, and stained with hematoxylin and eosin (H&E, Sigma-Aldrich Co., St. Louis, Missouri, United States). Histopathological features of these sections were observed by light microscopy, after which thicknesses of the mucosa and muscular layer were measured using the Leica Application Suite (Leica Microsystems, Heerbrugg, Switzerland).

### Analysis of Fecal Microbiota

After collection of fecal samples, total DNA of fecal microbiota was extracted from the samples by using a DNeasy PowerSoil Kit (Qiagen, Hilden, Germany) according to the manufacturer’s instructions. The extracted DNA was quantified using Quant-IT PicoGreen (Invitrogen, Carlsbad, CA, United States). Sequencing libraries were prepared according to the Illumina 16S Metagenomic Sequencing Library protocols used to amplify the V3 and V4 regions. The input gDNA 2 ng was PCR amplified with 1× reaction buffer, 1 nm of dNTP mix, 500 nm each of the universal F/R PCR primers, and 2.5 U of Herculase II fusion DNA polymerase (Agilent Technologies, Santa Clara, CA, United States). The cycle condition for the 1st PCR was 3 min at 95°C for heat activation, and 25 cycles of 30 s at 95°C, 30 s at 55°C, and 30 s at 72°C, followed by a 5-min final extension at 72°C. The universal primer pair with the Illumina adapter overhang sequences used for the first amplification were as follows: V3-F: 5'-TCGTC GGCAG CGTCA GATGT GTATA AGAGA CAGCC TACGG GNGGC WGCAG-3', V4-R: 5'-GTCTC GTGGG CTCGG AGATG TGTAT AAGAG ACAGG ACTAC HVGGG TATCT AATCC-3'. The first PCR product was purified with AMPure beads (Agencourt Bioscience, Beverly, MA). Following purification, 2 μl of the first PCR product was PCR amplified for final library construction containing the index using NexteraXT Indexed Primer. The cycle condition for the second PCR was similar to the first PCR condition except for 10 cycles. The PCR product was then purified with AMPure beads, and the final purified product was then quantified using qPCR according to the qPCR Quantification Protocol Guide (KAPA Library Quantification kits for Illumina Sequencing platforms) and qualified using the TapeStation D1000 ScreenTape (Agilent Technologies, Waldbronn, Germany). The paired-end (2 × 300 bp) sequencing was performed by the Macrogen unit using the MiSeq^™^ platform (Illumina, San Diego, CA, United States).

MiSeq raw data were curated using the Fastp program (Chen et al., 2018) and then assigned to operational taxonomic units (OTUs) using Cluster Database at High Identity with Tolerance (CD-HIT-OUT; Li et al., 2012). Based on BLAST+ (v2.9.0; Zhang et al., 2000) in the Reference DB (NCBI 16S Microbial), a representative sequence of each OTU was aligned. A comparative analysis of various microbial clusters was performed using QIIME (v1.9; Caporaso et al., 2010) with the above OTUs’ abundance and taxonomy information. The α-diversity information was obtained using the Shannon index and verified by examining the rarefaction curve and Chao1 values. The β-diversity was determined using weighted/unweighted UniFrac distance, and the flexibility was visualized *via* PCoA (Caporaso et al., 2010).

### FMT Analysis

The FMT analysis was performed as described in a previous study (Cao et al., 2017). Briefly, fresh fecal samples (10 g) were collected from 16-week-old male WT and C3 KO mice for FMT analysis and suspended with a sterile 0.2 ml of 1 × PBS solution to prepare a fecal mixture. After centrifugation, the suspensions were filtered with 0.45 μm of syringe filter and stored at −70°C in Eppendorf tubes until assayed.

**Figure 1:**
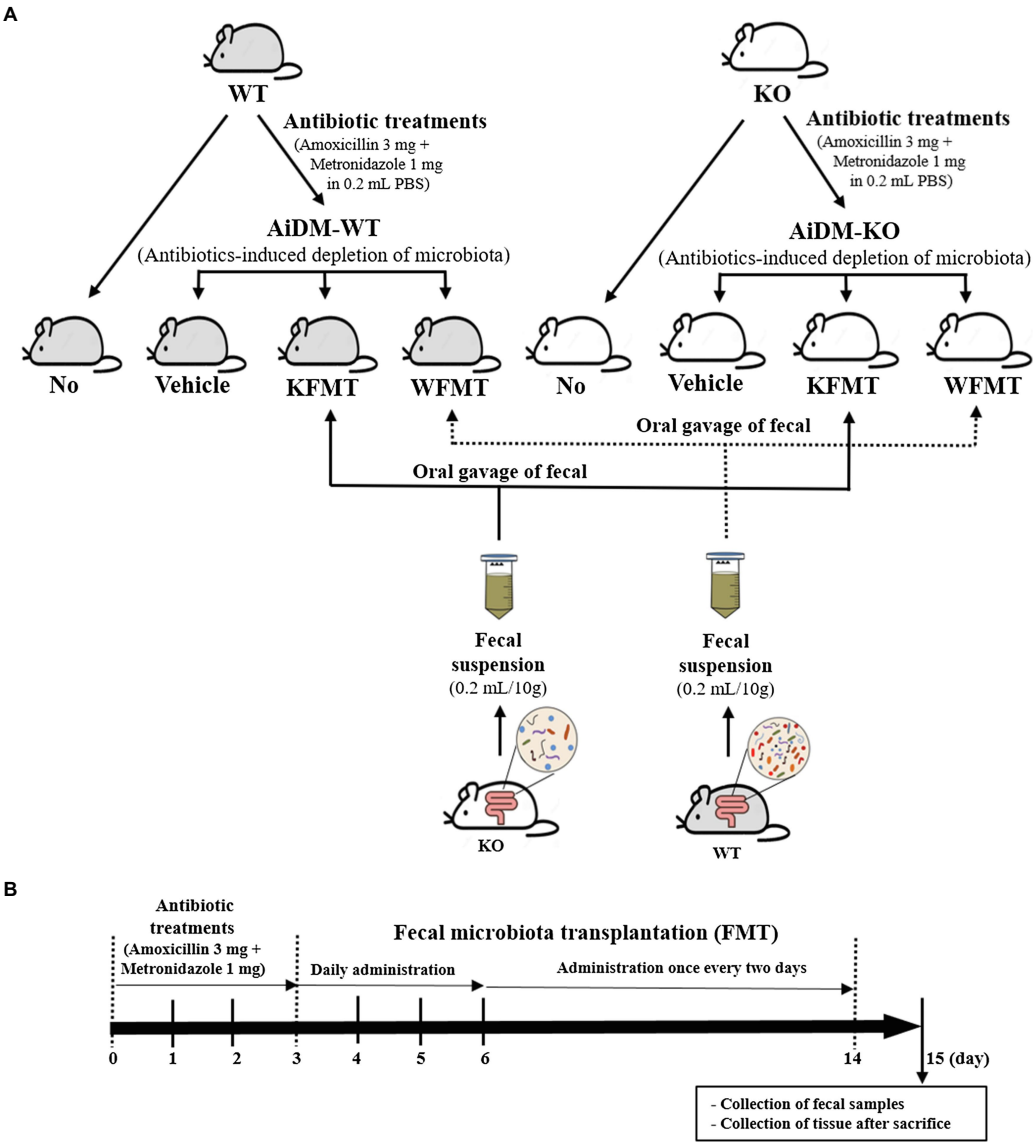
Experimental scheme for fecal microbiota transplantation (FMT). **(A)** Treated groups. WT and KO mice were classified into four different groups: No, Vehicle, KFMT, and WFMT groups. **(B)** Treatment schedule. After antibiotics treatment for 3 days, AiDM models received fecal suspensions of WT or KO mice seven times over 2 weeks. WT, wild type; KO, knockout; FMT, fecal microbiota transplantation; KFMT, knockout mice FMT; WFMT, wild-type mice FMT; AiDM, antibiotics-induced depletion of microbiota; AiDM-WT, AiDM-wild-type mice; and AiDM-KO, AiDM-knockout mice.

Next, each 16-week-old male WT (*n* = 28) or C3 KO mouse (*n* = 28) was assigned to either a non-treated group (No group, *n* = 7) or an antibiotics-treated group (*n* = 21). To produce the antibiotics-induced depletion of microbiota (AiDM) model, a mixture of amoxicillin (3 mg, Zoetis Inc., NJ, United States) and metronidazole (1 mg, CJ Cheil Jedang Inc., Seoul, Korea) in a 1 × PBS solution was daily treated into WT (AiDM-WT) and C3 KO (AiDM-KO) mice for 3 days by gavage, whereas the No group received 1 × PBS alone under the same pattern. AiDM-WT and AiDM-KO mice were divided into a Vehicle-treated group (*n* = 7), WT fecal suspension-treated group (*n* = 7, WFMT group), or a C3 KO fecal suspension-treated group (*n* = 7, KFMT group). The suspension from the WT fecal samples (10 g) in 0.2 ml of a 1 × PBS solution was daily transplanted to the WFMT group of AiDM-WT and AiDM-KO mice by gavage for 3 days, while the suspension from the KO fecal samples (10 g) in 0.2 ml of a 1 × PBS solution was daily transplanted to the KFMT group of AiDM-WT and AiDM-KO mice by the same method. Subsequently, these suspensions were treated into the same group once every two days for 8 days. After final treatment, mice of each subset group were placed in metabolic cages and bred for 24 h. At 9 a.m. on the fifteenth day, total stool, urine, water, and food were collected from the metabolic cage of each group, and the various levels were measured using appropriate methods. All mice in the No-WT and AiDM-WT (Vehicle, KFMT, and WFMT) as well as the No-KO and AiDM-KO (Vehicle, KFMT, and WFMT) groups were then euthanized using CO_2_ gas, after which mid-colon samples were acquired and stored at −70°C in Eppendorf tubes until assayed (Figure 1).

### Culture of Fecal-Derived Microbiota

Microorganisms in the FMT analysis of fecal samples of WT and C3 KO mice were investigated by using the viable cell count method. After the feces collected from mice of a subset group were serially diluted with sterile water, each diluent was inoculated on a nutrient agar plate composing peptone (0.5%), beef extract/yeast extract (0.3%), agar (1.5%), and NaCl (0.5%), and then cultured under aerobic condition at 30°C for 24 h. The number and pattern of colonies grown in the nutrient agar plate were counted and analyzed on the view box.

### Measurement of Intestinal Length

The intestinal length was measured by applying a method described previously (Choi et al., 2014). Briefly, all mice in the FMT analysis group were fasted for 18 h before the experiment but were allowed to consume water *ad libitum*. Mice were euthanized using CO_2_, and the intestinal tract was collected from the abdominal cavity. The total intestinal length from stomach to anus was measured in duplicate.

### Quantitative Real-Time PCR Analysis

Quantitative Real-Time PCR analysis (RT-qPCR) was applied to assess the relative quantities of AQP3, C-kit, and 5-HT mRNAs. Total RNA molecules were isolated from frozen mid-colon tissues using RNA Bee solution (Tet-Test Inc., Friendswood, TX, United States). After quantification of the RNA concentration, complement DNA (cDNA) was synthesized using a mixture of oligo-dT primer (Thermo Fisher Scientific Inc., MA, United States), dNTP, and reverse transcriptase (Superscript II, Thermo Fisher Scientific Inc.). RT-qPCR was then conducted using a cDNA template and 2 × Power SYBR Green (Toyobo Co., Osaka, Japan), as described in a previous study (Bae et al., 2020). The primer sequences used to evaluate mRNA levels were as follows: AQP3, sense primer 5'-GGTGG TCCTG GTCAT TGGAA-3', antisense primer 5'-AGTCA CGGGC AGGGT TGA-3'; C-kit, sense primer 5'-TGTTG CCTTC ACGGT TTTCC-3', antisense primer 5'-AACGA TCACT TCTTC CAGGT TCA-3'; 5-HT, sense primer 5'-CTGAG GCCCT CCCAC ATCT-3', antisense primer 5'-GGAAA GGAAC AAGGC CAACA-3'; β-actin, sense and antisense primers 5'-ACGGC CAGGT CATCA CTATT G-3' and 5'-CAAGA AGGAA GGCTG GAAAA GA-3', respectively. The thermal cycling conditions for DNA amplification consisted of holding stage (as 1 min at 95°C), cycling stage (40 cycles of 15 s at 95°C, 15 s at 57°C, and 45 s at 72°C), and melt curve stage (15 s at 95°C and 60 s at 60°C). Fluorescence intensity was measured at the end of the extension phase of each cycle. The threshold values for the fluorescence intensities of all samples were set manually. The reaction cycle at which the PCR products exceeded this fluorescence intensity threshold during the exponential phase of PCR amplification was considered the threshold cycle (Ct). Expression of the target gene was quantified relative to that of the housekeeping gene β-actin based on a comparison of the Cts at a constant fluorescence intensity and applying the Livak and Schmittgen method (Livak and Schmittgen, 2001).

### Statistical Analysis

Statistical significance was evaluated by performing one-way ANOVA (SPSS for Windows, Release 10.10, Standard Version, Chicago, IL, United States) followed by Tukey’s *post-hoc t*-test for multiple comparisons. Data are presented as mean ± standard deviation (SD) values, and *p* < 0.05 is considered to indicate a statistically significant difference.

## Results

### Induction of Constipation Phenotypes in C3 KO Mice

**Figure 2:**
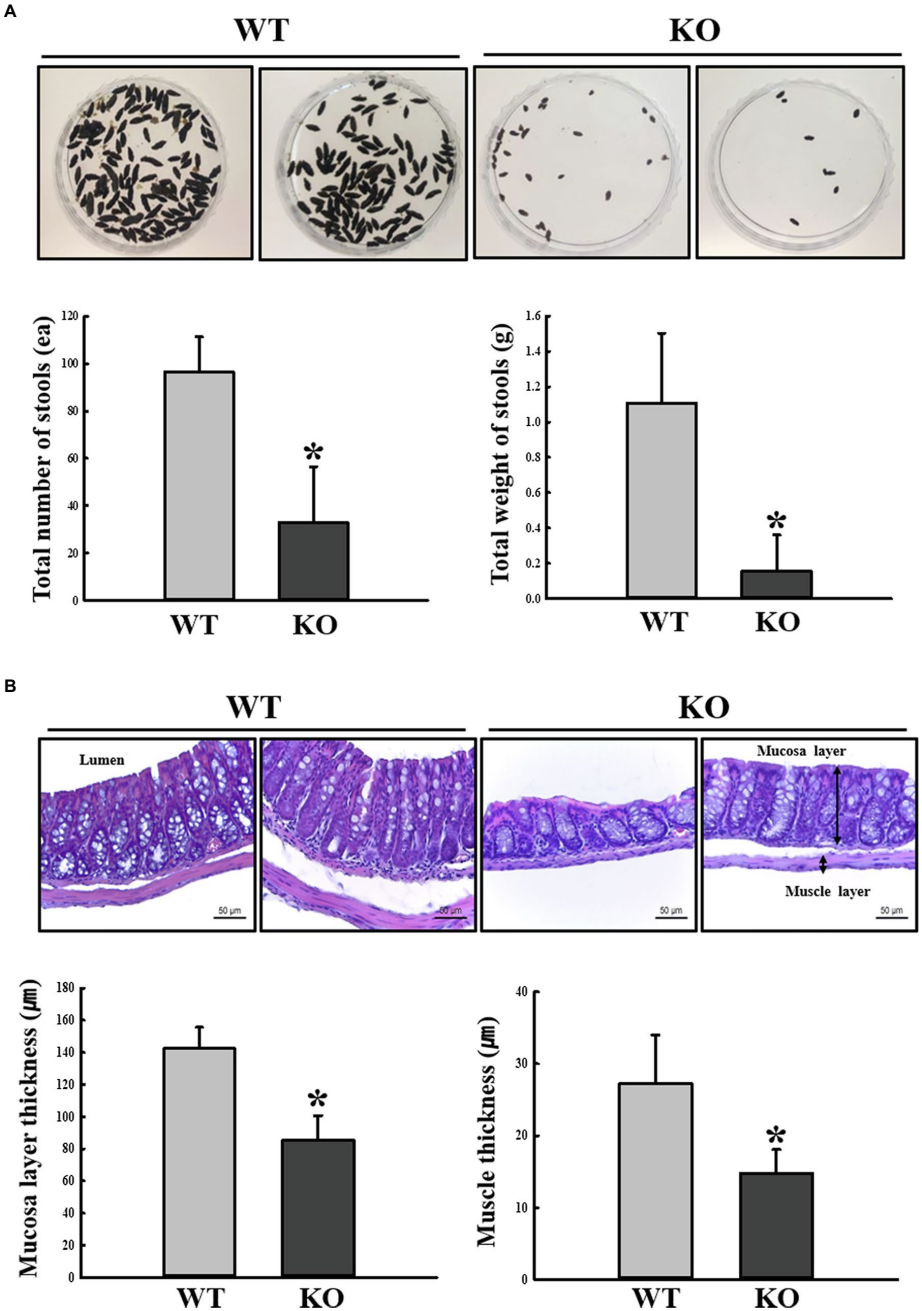
Stool parameters and histopathological structures of mouse mid-colon. **(A)** Digital camera images of stools were taken immediately after collection from a metabolic cage. Total number and weight of stools were measured as described in Materials and Methods. Three to five mice per group were used for stool collection, and each parameter was assayed in duplicate. **(B)** H&E-stained sections of mid-colon from WT and KO mice were observed at 400x magnification using a light microscope. Histopathological parameters were determined using the Leica Application Suite. Three to five mice per group were used to prepare H&E-stained slides, and histopathological parameters were measured in duplicate in each slide. The data are reported as mean ± SD values. ^*^p < 0.05 compared with the WT group. WT, wild type; KO, knockout; and H&E, hematoxylin and eosin.

First, we confirmed the induction of constipation phenotypes in C3 KO mice based on the method described in a previous study (Park et al., 2021). To achieve this, alterations of fecal parameters and histopathological structure of mid-colons were examined in 16-week-old C3 KO mice. A significant decrease in the number and weight of fecal samples was detected in the C3 KO mice compared with those of WT mice (Figure 2A). In the histopathological analysis of the mid-colon, thicknesses of the mucosal layer and muscle were remarkably decreased in C3 KO mice (Figure 2B). However, the levels of three parameters for feeding behavior were constantly remained in the WT and C3 KO groups, respectively (data not shown). The above results suggest that constipation phenotypes could be successfully detected in 16-week-old C3 KO mice, allowing the investigation of dysbiosis of fecal microbiota.

### Alteration in the Profile of the Fecal Microbiota of C3 KO Mice

**Figure 3:**
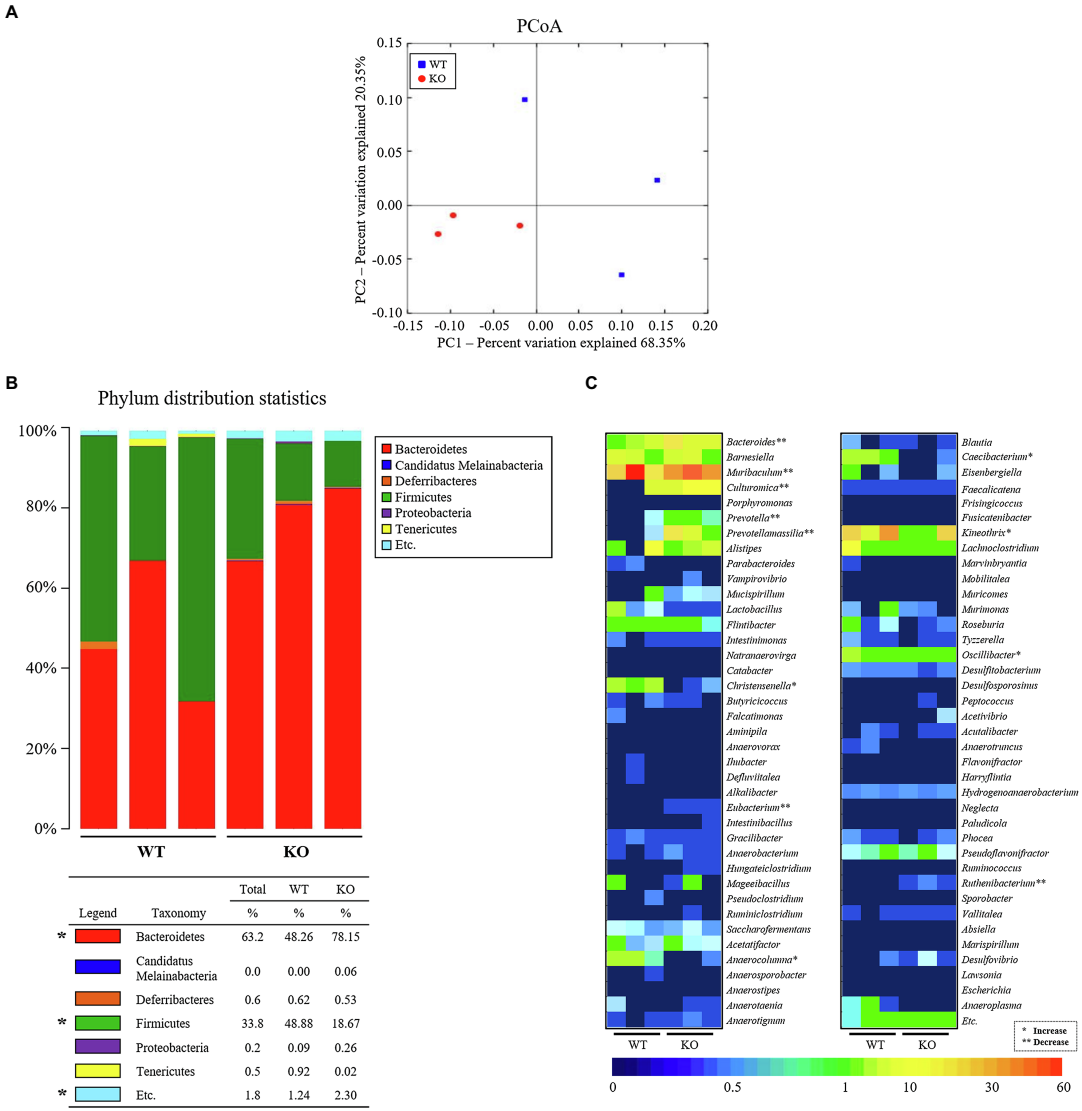
Characterization of fecal microbiota. **(A)** PCoA plot analysis. The PCA focused on fecal bacterial communities using the principle components in WT and C3 KO mice. The spatial distance measure indicates the degree of similarity of bacterial taxa in the fecal sample. WT: blue rectangle and KO: red circle. **(B)** Fecal microbiota distribution at the phylum level in WT and KO mice. ^*^p < 0.05 compared with the WT group. **(C)** Heat map showing a significant difference between WT and KO mice at the bacteria genus level. Different colors indicate the relative abundance of each genus. WT, wild type; KO, knockout; PCoA, principal coordinate analysis; and PCA, principal component analysis.

To investigate whether the profile of the fecal microbiota of C3 KO mice is affected by C3 deficiency-induced constipation development, overall microbial composition was analyzed in fecal samples of WT and C3 KO mice. The results of a principal component analysis plot exhibited that the fecal microbial community of C3 KO mice was significantly different from that of WT mice. Diversity of the microbial population was lower in C3 KO mice than in WT mice (Figure 3A). Analysis of the microbial phyla showed the population of *Bacteroidetes* was increased (by 61.9%) in C3 KO mice compared with that in WT mice, while the Firmicutes population was remarkably decreased (by 61.8%) in the same group (Figure 3B). Furthermore, a significant decrease at the genus level was detected in *Anaerocolumna*, *Caecibacterium*, *Christensenella*, *Kineothrix*, and *Oscillibacter* present in the fecal samples of C3 KO mice. The largest increase at the genera level in C3 KO mice was observed for *Anaerocolumna* (97%), followed by *Caecibacterium* (97%), *Christensenella* (93%), *Kineothrix* (62%), and *Oscillibacter* (45%; Figure 3C). The proportions of various microbial genera, including *Prevotellamassilia*, *Reuthenibacterium*, *Prevotella*, *Eubacterium*, *Culturomica*, *Bacteroides*, and *Muribaculum*, were increased in C3 KO mice. The largest decrease in the microbial genera in C3 KO mice was observed for *Prevotellamassilia* (48.4-fold), followed by *Reuthenibacterium* (14-fold), *Prevotella* (6.3-fold), *Eubacterium* (4.6-fold), *Culturomica* (2.6-fold), *Bacteroides* (1.7-fold), and *Muribaculum* (0.2-fold; Figure 3C). Among 12 microbial genera, the nine most altered microbial genera were Gram-negative bacteria, with only three of the genera being Gram-positive bacteria. These results indicate that C3 deficiency-induced constipation development may be closely linked to dysbiosis of the fecal microbiota in C3 KO mice.

### Effects of KFMT on Fecal Parameters and Growth of Fecal Microbiota in AiDM-WT and AiDM-KO Mice

**Figure 4:**
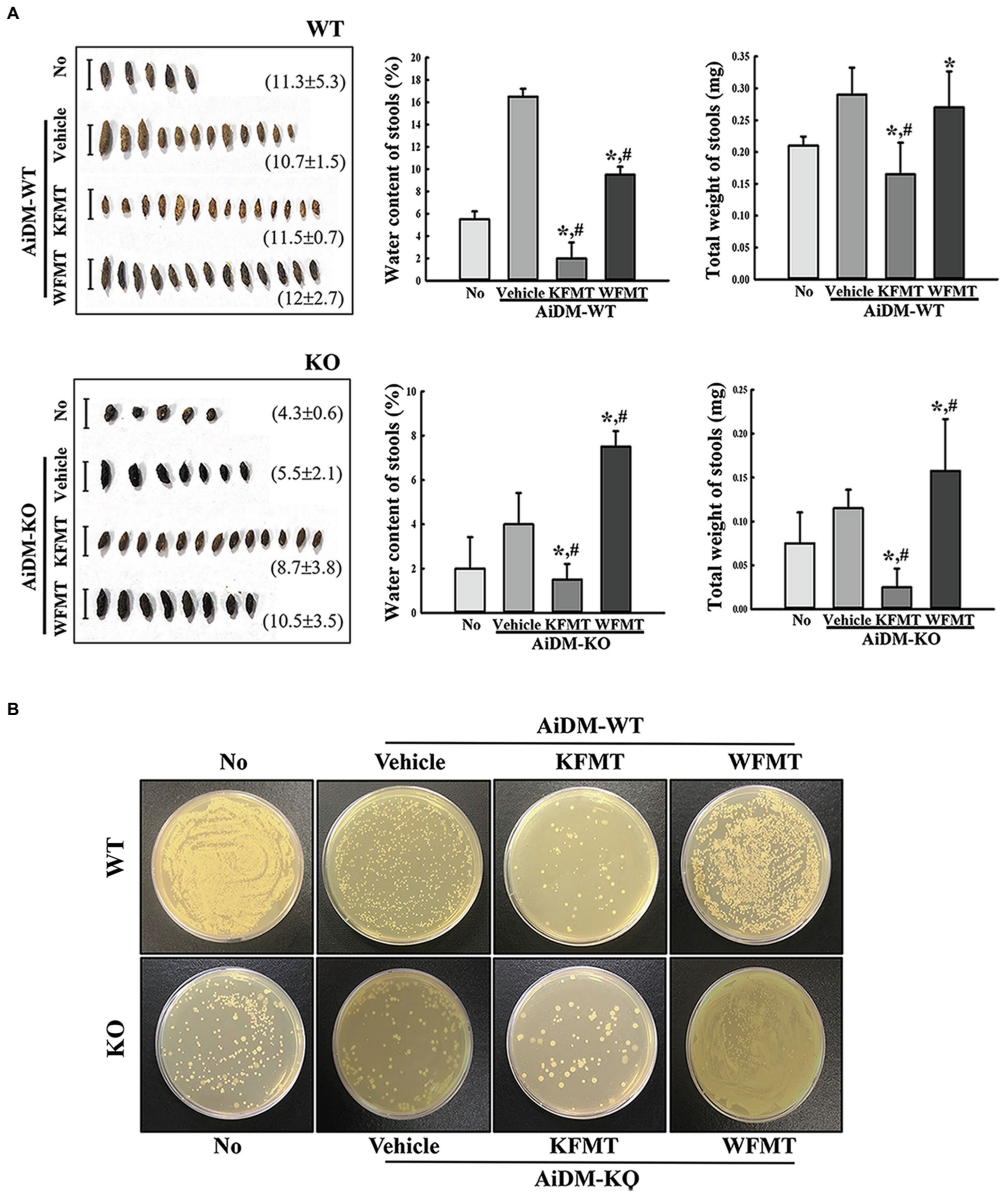
Stool parameters and fecal microbiota in FMT mice. **(A)** Stool morphological characteristics. Digital camera images of stools were taken immediately after collection from a metabolic cage. The total number and weight of stools were measured as described in Materials and Methods. Stool water content was calculated by comparing the weight of fresh stool and dried stool weight. **(B)** Cultivation of fecal microbiota. Microorganism colonies formed on the nutrient agar were observed in a view box. Three to five mice per group were used for stool collection, and each parameter was assayed in duplicate. The data are reported as mean ± SD values. ^*^p < 0.05 compared with the No-treated group. ^#^*p* < 0.05 compared with the AiDM+Vehicle-treated group. WT, wild type; KO, knockout; FMT, fecal microbiota transplantation; KFMT, knockout mice FMT; WFMT, wild-type mice FMT; AiDM, antibiotics-induced depletion of microbiota; AiDM-WT, AiDM-wild-type mice; and AiDM-KO, AiDM-knockout mice.

Next, we examined whether the fecal microbiota altered by C3 deficiency-induced constipation development can be induced the defecation delay by performing FMT study. To achieve this, fecal suspensions of WT and C3 KO mice were administrated to AiDM-WT and AiDM-KO mice, and subsequently, fecal parameters and growth of fecal microbiota were analyzed. Water content and total weight of fecal were significantly decreased in the KFMT group compared to those of the WFMT group of AiDM-WT and AiDM-KO mice, although the alteration pattern of these parameters was not reflected by the changes in fecal microbiota abundance. Also, the shape of the fecal samples was smaller, thinner, and harder in KFMT group than in the WFMT group (Figure 4A). Furthermore, these alterations in fecal parameters were wholly reflected by the growth patterns of the fecal-derived microbiota in nutrient agar plates. The number of bacterial colonies was lower in the KFMT group than in the other subset groups of AiDM-WT mice but was higher in the WFMT group than in the other subset groups of AiDM-KO mice (Figure 4B). These results suggest that the alteration of the fecal microbiota derived from C3 KO mice with constipation phenotypes may be closely correlated with defecation delay effects and dysbiosis of intestinal microbiota in AiDM mice.

### Effects of KFMT on Intestinal Length in AiDM-WT and AiDM-KO Mice

**Figure 5:**
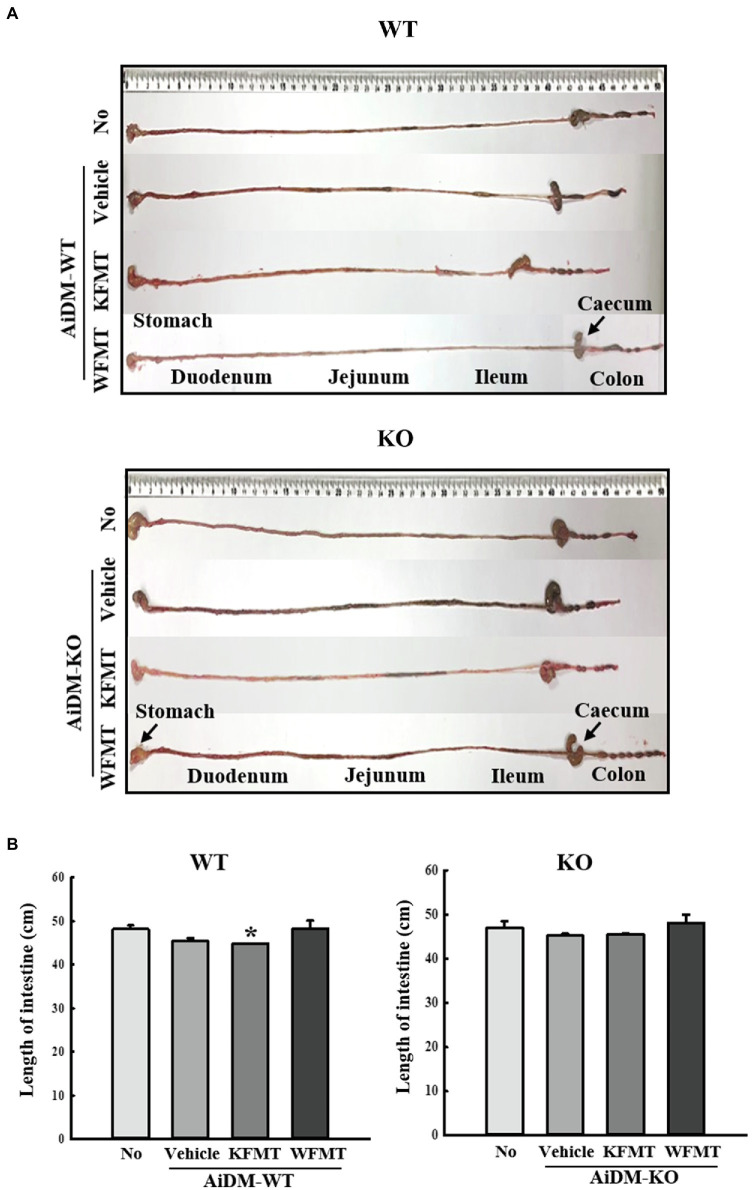
Intestinal length in FMT mice. **(A)** Representative image showing an entire intestine. The total intestinal tract was excised from a mouse of each subset group. Morphology was observed using a digital camera. **(B)** Total length of intestine. The total length from stomach to anus was measured with a ruler. Three to five mice per group were used in the collection of intestines, and the intestine length was measured twice. The data are reported as mean ± SD values. ^*^p < 0.05 compared with the No-treated group. WT, wild type; KO, knockout; FMT, fecal microbiota transplantation; KFMT, knockout mice FMT; WFMT, wild-type mice FMT; AiDM, antibiotics-induced depletion of microbiota; AiDM-WT, AiDM-wild-type mice; and AiDM-KO, AiDM-knockout mice.

To investigate whether KFMT-induced defecation delay effects are accompanied by alterations in intestinal length, we measured intestine lengths in AiDM-WT and AiDM-KO mice after WFMT and KFMT. Total intestine length was significantly decreased only in the KFMT group compared with that of the other subset group of AiDM-WT mice. However, these alterations were not observed in the KFMT and WFMT groups of AiDM-KO mice (Figure 5). These results suggest that KFMT-induced defecation delay effects are associated with regulating the intestinal length in AiDM-WT mice although the delay did not affect AiDM-KO mice.

### Effects of KFMT on the Histopathological Structure of the Mid-Colon in AiDM-WT and AiDM-KO Mice

**Figure 6:**
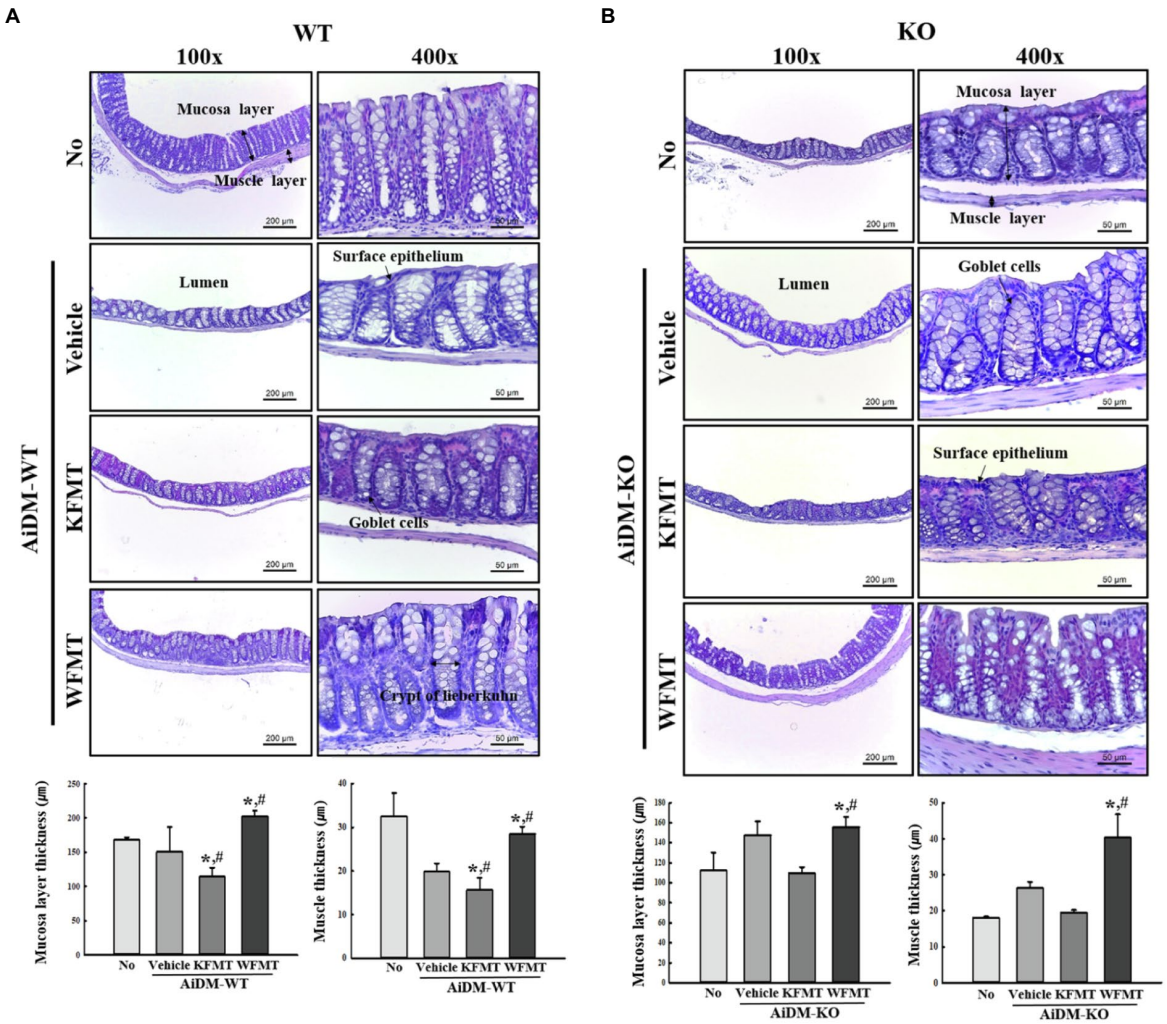
Histopathological structures of the mid-colon in FMT mice. H&E-stained sections of mid-colons from the No-, AiDM+Vehicle-, AiDM+KMFT-, or AiDM+WFMT-treated groups in WT **(A)** and KO **(B)** mice were observed at 40× (left column) and 400× (right column) using a light microscope. Histopathological parameters were determined using the Leica Application Suite. Four to six mice per group were used to prepare H&E-stained slides, and the histopathological parameters were measured in duplicate in each slide. The data are reported as mean ± SD values. ^*^p < 0.05 compared with the No-treated group. ^#^*p* < 0.05 compared with the AiDM+Vehicle-treated group. WT, wild type; KO, knockout; FMT, fecal microbiota transplantation; KFMT, knockout mice FMT; WFMT, wild-type mice FMT; AiDM, antibiotics-induced depletion of microbiota; AiDM-WT, AiDM-wild-type mice; and AiDM-KO, AiDM-knockout mice.

To investigate changes in the histological structure of the mid-colon associated with the KFMT-induced defecation delay effects in the AiDM model, we examined alterations in the histological parameters indicating constipation in H&E-stained mid-colons of the subset groups. In AiDM-WT mice, thicknesses of the mucosa layer and muscle were significantly decreased in KFMT group compared with those of the Vehicle or No groups, whereas the thicknesses were increased or constant in WFMT group of AiDM-WT mice. Also, significant changes, including irregular shape, unequal distribution, and various sizes of goblet cells, were observed in the crypts of Lieberkuhn in the mid-colon sections of the KFMT group (Figure 6A). Meanwhile, a different pattern was detected in the histological structure of the mid-colon in AiDM-KO mice. The thicknesses of the mucosa layer and muscle were slightly enhanced in Vehicle group compared with those of the No groups, while these levels were constantly remained in KFMT group. But, WFMT group showed an increase level compared with No or Vehicle group (Figure 6B). These results indicate that KFMT-induced defecation delay effects contribute to structural abnormalities in the mid-colon of AiDM-WT, but not of AiDM-KO mice.

### Effects of KFMT on the Molecular Regulators of Constipation in the Mid-Colon of AiDM-WT and AiDM-KO Mice

**Figure 7:**
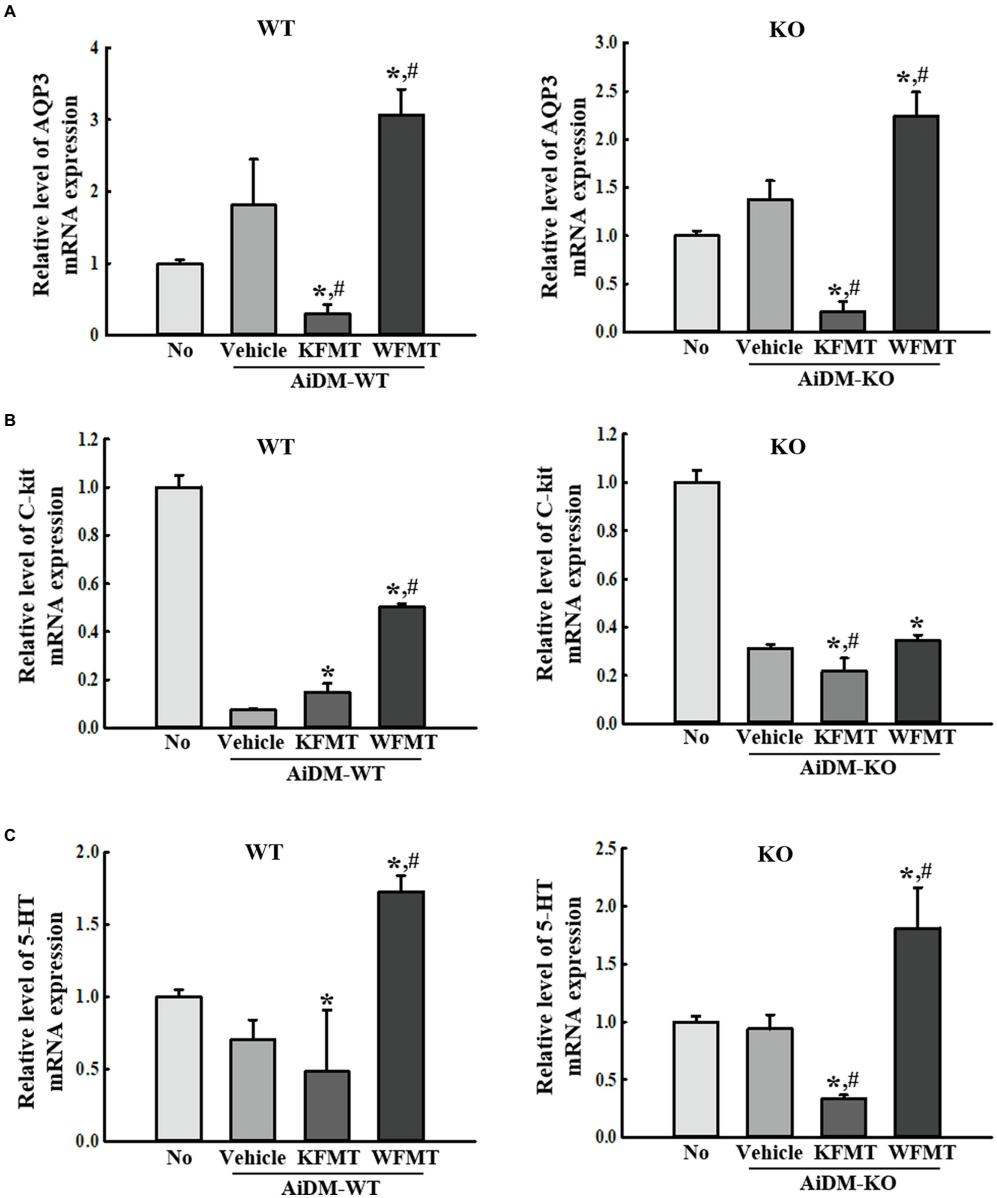
Molecular regulator expressions in FMT mice. **(B)** RT-qPCR analyses of AQP3 **(A)**, C-kit **(B)**, and 5-HT **(C)**. The levels of AQP3, C-kit, and 5-HT transcripts in the total mRNA of mid-colons were measured by performing RT-qPCR with specific primers. The mRNA levels of the three genes were based on the intensity of actin as an endogenous control. Three to five mice per group were used to prepare total RNA, and RT-qPCR analyses were performed in duplicate for each sample. The data are reported as mean ± SD values. ^*^p < 0.05 compared with the No-treated group. ^#^*p* < 0.05 compared with the AiDM+Vehicle-treated group. WT, wild type; KO, knockout; FMT, fecal microbiota transplantation; KFMT, knockout mice FMT; WFMT, wild-type mice FMT; AiDM, antibiotics-induced depletion of microbiota; AiDM-WT, AiDM-wild-type mice; and AiDM-KO, AiDM-knockout mice.

Finally, we investigated whether KFMT-induced defecation delay effects are accompanied by alterations in the molecular regulators for constipation, including AQP3, C-kit, and 5-HT, because their roles are associated with the regulation of water content, proliferation of interstitial cells of Cajal, and gastrointestinal mobility during constipation. To achieve this, the expression level of AQP3, C-kit, and 5-HT was measured in the mid-colon of AiDM-WT and AiDM-KO mice after WFMT and KFMT. Similar regulation patterns were observed for AQP3 and 5-HT expressions. These levels were significantly decreased only in the KFMT group of AiDM-WT and AiDM-KO mice, whereas the WFMT group of AiDM-WT and AiDM-KO mice showed enhanced levels of AQP3 and 5-HT (Figures 7A,C). Meanwhile, the expression level of C-kit was lower in the Vehicle group than in the No group of AiDM-WT and AiDM-KO mice. However, in AiDM-WT mice, this level was increased in both KFMT and WFMT groups, although the rate of increase was greater in the WFMT group than in the KFMT group. In AiDM-WT mice, a slight decrease in the expression level of C-kit was observed in KFMT group (Figure 7B). The above results indicate that KFMT-induced defecation delay effects may be associated with the regulation of AQP3, C-kit, and 5-HT expressions in the mid-colons of AiDM-WT and AiDM-KO mice.

## Discussion

Until now, there has been abundant scientific evidence supporting the hypothesis that an alteration of the microbiota composition in the gut is closely associated with various chronic diseases, including cardiovascular disease, obesity, metabolic syndrome, IBDs, brain injury, and Alzheimer’s disease (Hand et al., 2016; Kowalski and Mulak, 2019). Among these diseases, chronic constipation could be the result of dysfunction of gastrointestinal mobility induced by alteration of the gut’s microbiota composition (Quigley, 2011; Zhu et al., 2014). During constipation, intestinal microbiota has an important role in the regulation of gastrointestinal mobility, tissue repair, maturation of gut-associated lymphoid tissue, and barrier function (Sommer and Backhed, 2013). In this study, we investigated the function and action mechanism of dysbiosis on the fecal microbiota of mice with C3 deficiency-induced constipation by performing microbiota composition analysis and FMT experiments. Our results showed that significant alterations of the profile of fecal microbiota could be detected in mice with C3 deficiency-induced constipation. Also, they suggested that the dysbiosis of fecal microbiota derived from constipated C3 KO mice can successfully induce defecation delay effects in AiDM mice.

The possibility of a correlation between C3 regulation and intestinal diseases has been suggested by several sources of evidence in previous studies. The small intestinal crypts in Crohn’s disease (CD) patients show a significant alteration in the level of C3 and C4 mRNA, while the intestinal mucus of IBD patients exhibits upregulated levels of C3 mRNA (Laufer et al., 2000; Sugihara et al., 2010). Also, the concentrations of C3 and a C3 fragment were increased in the jejunal secretions of CD patients as well as in the epithelia of the small and large intestines in CD patients (Ahrenstedt et al., 1990; Halstensen et al., 1992; Riordan et al., 1996). Furthermore, these products have been detected in the mucus of ulcerative colitis patients and the colonic mucosa of dextran sodium sulfate-treated mice (Ueki et al., 1996; Lin et al., 2004). Additional evidence of an association was provided by our previous study that was the first to detect constipation phenotypes in CRISPR/Cas9-mediated C3 KO mice (Park et al., 2021). In that study, stool excretion parameters, gastrointestinal transit, and intestine length were shown to be remarkably decreased in C3 KO mice compared with those in WT mice; moreover, the histopathological structure and mucin secretion levels were significantly changed in the mid-colon of C3 KO mice. Therefore, the present study was conducted to provide results complementary to those of the previous study. The present results provide novel scientific evidence that dysbiosis of fecal microbiota can be considered an important factor in C3 deficiency-induced constipation.

Differences in the population diversity of intestinal microorganisms in patients with constipation have been observed in previous studies (Simren et al., 2013). The differences may be the result of the application of different study methods, use of single samples from patients with constipation, and the variety of diets they ingested. Fecal microbiota has been assessed in many studies because samples are easily collected and may be simply analyzed. However, the results of such studies have not fully reflected the profile of mucosal microbes in patients with constipation. Moreover, mucosal microbes are more abundant and have a greater influence on the regulation of epithelial and mucosal function than that of fecal microbiota (Durban et al., 2012; Parkes et al., 2012). Correlations between mucosal and fecal microbiota have been investigated in female patients with constipation (Parthasarathy et al., 2016). In the present study, fecal samples collected from mice in metabolic cages were used to examine the population diversity and fecal microbiota profiles in C3 KO mice. The present study provides only limited information regarding the intestinal microbiota of C3 KO mice with constipation. Further research using mucosal tissue of C3 KO mice is required to elucidate the overall composition of the colonic mucosal microbiota and verify the critical role of dysbiosis in C3 deficiency-induced constipation.

In the present study, significant alterations in the microbial population were observed in 12 microbial genera. Among these, five microbial genera showed remarkable decreases in constipated C3 KO mice, whereas seven of those genera increased in abundance in the same group. However, in most previous constipation studies, alterations in the microbial population were observed in only a few microbial genera. Only three microbial genera (*Prevotella*, *Bifidobacterium*, and *Lactobacillus*) were observed to be decreased in constipated children (Khalif et al., 2005; Zhu et al., 2014), whereas five genera (*Bifidobacterium*, *Clostridia*, *Cerococcids*, *Roseburia*, and *Faecalibacterium*) increased in constipated children and patients (Zoppi et al., 1998; Pryde et al., 2002). The largest number of changes in microbial genera during constipation was reported by Gerritsen et al. (2011). Constipated patients showed increases in two genera (*Pseudomonas* and *Campylobacter*) and decreases in three genera (*Lactobacillus*, *Bifidobacterium*, and *Bacteroides*; Gerritsen et al., 2011). The difference between the results of our study and previous studies is thought to be due to differences in the target species used in the experiments, as there are many differences in the food and environment aspects of humans and mice. Also, differences in the cause of the observed constipation are expected to significantly affect the number of altered microbe genera.

Regardless, *Prevotella* and *Bacteroides* are commonly reported to be at decreased levels in both humans and C3 KO mice during constipation, although the changes are different. The levels of those two genera decreased in constipated human patients but increased by 6.3-fold and 1.7-fold, respectively, in the fecal microbiota of C3 KO mice (Zhu et al., 2014; Kim et al., 2015). *Prevotella* is an anaerobic Gram-negative bacterium that is naturally distributed in oral cavity of animals (Tanaka et al., 2008). In the gut, this genus, acting as a producer of anti-inflammatory metabolites, decreases Th17 polarization and stimulates the differentiation of Treg/Tr1 cells with anti-inflammatory activity (Li et al., 2016). *Bacteroides* is a predominant genus in the lower part of the intestinal tract and is abundantly distributed in fecal matter (Salyers, 1984; Patrick, 2015). This microbial genus maintains a beneficial relationship with the host organism but can induce abscess formation in the various sites within the body as well as bacteremia (Wexler, 2014). However, there are no reports describing the roles of *Prevotella* and *Bacteroides* in the initiation and progression of constipation, until now. Our results provide novel evidence that *Prevotella* and *Bacteroides* are likely to be useful as one of the candidates for indicators of constipation in humans and mice, although further studies and evaluations are needed.

In this study, we identified 12 genera that showed significant changes in relative abundance in the fecal microbiota of C3 KO mice. Among these, nine genera (*Anaerocolumna*, *Caecibacterium*, *Christensenella*, *Kineothrix*, *Oscillibacter*, *Prevotellamassilia*, *Reuthenibacterium*, *Eubacterium*, and *Culturomica*) were first identified in this study as microbiota members that may be altered in abundance during constipation. The genus *Prevotellamassilia*, which showed the highest rate of increase in this study, was recently isolated from a fecal specimen of a melanoma patient (Ndongo et al., 2016). The genus *Eubacterium* with a 4.6-fold increase in C3 KO mice is an anaerobic Gram-positive bacterium widely present in the oral area and the intestinal tract of human and animals, as well as in soil (Wade, 2006). The genus *Anaerocolumna*, which was shown to be completely inhibited in C3 KO mice, is an anaerobic Gram-positive bacterium that has been isolated from human fecal, soil, and fermented food samples (Ueki et al., 2016). Also, the alteration pattern of the genus *Caecibacterium*, an anaerobic Gram-negative bacterium, was very similar to that of *Anaerocolumna* (Onrust et al., 2017). However, no significant evidence of the regulation of bowel function by the above nine genera has been reported. Regardless, our results suggest the possibility that nine genera identified in C3 KO mice may have important roles in constipation-related diseases. The other three genera have been previously reported to be related to constipation. The genera *Prevotella* and *Bacteroides* were reported to be significantly decreased in a fecal sample of a constipated patient compared with that of a normal sample (Gerritsen et al., 2011; Zhu et al., 2014). Interestingly, there were reverse patterns for the above two genera in the fecal samples of C3 KO mice. Also, the genus *Muribaculum* has been reported to be decreased in fecal samples of water limitation-induced constipated models (Liu et al., 2017). In contrast, there was a significant increase detected in the C3 KO mice. *Muribaculum* was first identified as a Gram-negative bacterium from the gut of mouse and the intestines of other animals (Lagkouvardos et al., 2019).

Meanwhile, there is little research into the correlation between microbiota dysbiosis and histological structure of intestine. In obstructive bowel disorder, the relative abundance of *Firmicutes* was decreased, while those of *Proteobacteria* and *Bacteroidetes* was increased compared with sham controls. Also, the inflammatory infiltration was increased in the histological structure of colon muscularis externae and colon smooth muscle contractility suppression (Gerritsen et al., 2011). In 5-Fluorouracil (5-FU)-induced intestinal mucositis of mice, a decrease of *Firmicutes* abundance and an increase of *Bacteroidetes* abundance were accompanied with shortening of villi and destruction of crypts (Kato et al., 2017). In rotenone-induced GI dysfunction model, the intestinal microbial diversity is slightly increased in the colon and reduced in the small intestine, while the amount of goblet cells and mucosal thickness is increased in colon (Johnson et al., 2018). In this study, we analyzed microbiota dysbiosis in C3 KO mice with constipation. A significant decrease on the mucosal and muscle layer thickness was accompanied with the alteration of two phyla, including *Bacteroidetes* and *Firmicutes*. Our study suggests the first evidence for the correlation between microbiota dysbiosis and histopathological structure of colon. Also, in the FMT study, a significant alteration on the histological structure of mid-colon was observed in AiDM-WT mice after KFMT, but AiDM-KO mice was returned to those of No treated group. However, there is no scientific basis for the clear causes of these differences. A variety of further studies will be needed to address this issue.

## Conclusion

In the present study, we investigated the effects of dysbiosis on the fecal microbiota of C3 KO mice during C3 deficiency-induced constipation. Taken together, our data indicate that C3 deficiency-induced constipation is associated with increases in seven genera of bacteria and decreases in five bacteria genera present in fecal microbiota. Also, the results of the present study suggest that dysbiosis of C3 KO mice with constipation phenotypes can induce defecation delay effects in a model with an antibiotics-induced reduction in the number of microbes. Furthermore, our results provide novel evidence of the potential for microbiome-based therapies using new therapeutic targets and treatment approaches to prevent and treat the patient with chronic constipation; however, further research into such therapy is needed.

## Data Availability Statement

The datasets presented in this study can be found in online repositories. The names of the repository/repositories and accession number(s) can be found at:

https://www.ncbi.nlm.nih.gov/, NM_016689.2

https://www.ncbi.nlm.nih.gov/, Y00864.1

https://www.ncbi.nlm.nih.gov/, X79283.1

https://www.ncbi.nlm.nih.gov/, NM_007393.5.

## Ethics Statement

The animal study was reviewed and approved by the Pusan National University-Institutional Animal Care and Use Committee (PNU-IACUC; approval number PNU-2019-2292).

## Author Contributions

DH designed and supervised this work and wrote the manuscript. YC and JK completed the experiments. YC, JK, SL, and JG evaluated and analyzed the results. HS and JH reviewed and edited the manuscript. All authors approved the final manuscript.

### Conflict of Interest

The authors declare that the research was conducted in the absence of any commercial or financial relationships that could be construed as a potential conflict of interest.

## Abbreviations

C3: complement component 3
FMT: fecal microbiota transplantation
AiDM: antibiotics-induced depletion of microbiota
WFMT: FMT of WT mice
KFMT: FMT of KO mice
PNU-IACUC: Pusan National University-Institutional Animal Care and Use Committee
FDA: Food and Drug Administration
CT: threshold cycle
IBD: inflammatory bowel disease
UC: ulcerative colitis
CD: Crohn’s disease
GALT: gut-associated lymphoid tissue
